# Measuring changes in self-concept: a qualitative evaluation of outcome questionnaires in people having acupuncture for their chronic health problems

**DOI:** 10.1186/1472-6882-6-7

**Published:** 2006-03-16

**Authors:** Charlotte Paterson

**Affiliations:** 1 MRC Health Services Research Collaboration, Department of Social Medicine, University of Bristol. Bristol, UK

## Abstract

**Background:**

Changes in self-concept are an important potential outcome for many interventions for people with long-term conditions. This study sought to identify and evaluate outcome questionnaires suitable for quantifying changes in self-concept in people with long-term conditions, in the context of treatment with acupuncture and Chinese medicine.

**Methods:**

A literature search was followed by an evaluation of three questionnaires: The Wellbeing Questionnaire W-BQ12, the Patient Enablement Instrument (PEI), and the Arizona Integrative Outcome Scale (AIOS). A convenience sample of 23 people completed the questionnaires on two occasions and were interviewed about their experience and their questionnaire responses. All acupuncturists were interviewed.

**Results:**

Changes in self-concept were common and emerged over time. The three questionnaires had different strengths and weaknesses in relation to measuring changes in self-concept. The generic AIOS had face validity and was sensitive to changes in self-concept over time, but it lacked specificity. The PEI was sensitive and specific in measuring these changes but had lower acceptability. The sensitivity of the W-BQ12 was affected by initial high scores (ceiling effect) and a shorter timescale but was acceptable and is suitable for repeated administration. The PEI and W-BQ12 questionnaires worked well in combination.

**Conclusion:**

Changes in self-concept are important outcomes of complex interventions for people with long-term illness and their measurement requires carefully evaluated tools and long-term follow-up. The literature review and the analysis of the strengths and weaknesses of the questionnaires is a resource for other researchers. The W-BQ12 and the PEI both proved useful for this population and a larger quantitative study is planned.

## Background

Relevant, comprehensive, and valid outcome measures are a pre-requisite for all experimental and observational evaluations of healthcare interventions. The choice of outcome measures is especially problematic in evaluating complex interventions, interventions for people with long-term health problems, and interventions that aim to bring about a wide range of treatment effects and behaviour change. In these situations it has been suggested that a staged process of enquiry should be instituted, and that qualitative and theoretical work may play an important part in developing research designs [1]. Qualitative methods are useful in understanding the wide range of treatment effects that may be valued in such situations; in linking these individual experiences to more theoretically based concepts; and in designing and evaluating outcome questionnaires that can be used to quantify these treatment effects. By raising descriptive qualitative findings to the level of concepts, such preliminary work may be transferable to other interventions and populations. This paper is based on a programme of primary research into evaluating acupuncture and Chinese medicine, but the wider issues of the definition and measurement of changes in self-concept are relevant to evaluating many other educational and healthcare interventions for people with long-term health problems.

Qualitative studies of people with long-term health problems that use acupuncture and Chinese medicine have identified a range of valued treatment effects and have started grouping them into categories and concepts [2-4]. In addition to changes in symptoms, medication, energy and relaxation, there are other changes that have been described by different researchers as 'improvement in psychosocial coping' [2] or 'changes in personal and social identity' [3]. There is considerable overlap in these two categories. Improvements in psychosocial coping included an increase in self-awareness, an increased sense of wholeness, balance, centredness, well-being, increases in self-efficacy and all round changes in lives; and the category of changes in personal and social identity included changes in self-awareness, self-acceptance, self-confidence, self-responsibility and self-help. In this paper the broad term of 'self-concept' is used to encompass this range of descriptors and the definition of the term 'self-concept' is explored as part of the literature search reported below. The significance of these changes in self-concept to some of the individuals studied is in accordance with a whole body of work that has investigated how people experience and adapt to the 'biographical disruption' of long-term illness (including survival from cancer) and the importance of re-negotiating and coming to terms with changing identities over time [5-7]. They are also of interest in relation to the use of social cognitive theory in health promotion interventions [8].

Previous research has suggested that changes in self-concept are not reliably measured by the generic outcome questionnaires EuroQol-5D and COOP-WONCA charts, or by the individualised problem-specific questionnaire Measure Yourself Medical Outcome Profile, MYMOP[9]. The study reported below aimed to identify and evaluate outcome questionnaires that would be suitable for quantifying changes in self-concept in people with long-term health problems who were having acupuncture and Chinese medicine for the first time. A literature search identified two questionnaires that were then evaluated, alongside a third brief generic questionnaire, using mixed methods. The method and results of the literature search are described first, followed by the details of the evaluation of the three questionnaires.

## Methods

The literature search is reported first and then the methods employed in evaluating the questionnaires.

### Literature search

**Figure 1:**
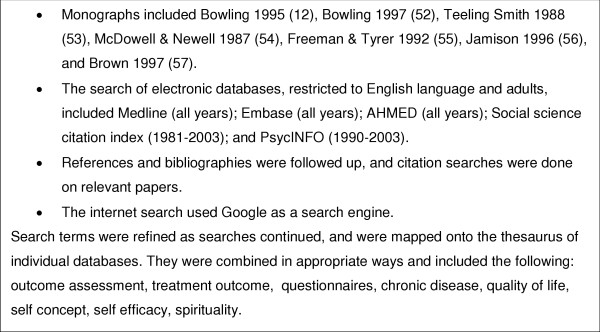
Literature review: sources and search terms.

This search took place in September/October 2003 and looked for previously used and validated questionnaires that would be suitable for piloting as outcome measures for acupuncture. It included papers that contributed to the meaning and definition of self-concept; and papers describing the use and validation of outcome questionnaires that sought to measure changes in self-concept in clinical settings. The sources and search terms are summarised in Figure 1. Relevant papers were used to widen and refine search terms, as sources of further references, and as a basis for citation searching. Authors were approached for copies of questionnaires of interest.

The decision about whether a questionnaire was likely to be suitable for piloting as an outcome measures for acupuncture was made on the basis of their relevance to concepts described in previous research work in this area; their availability as a written questionnaire; evidence of some validation work; and face validity in terms of acceptable length and language for people suffering from a range of long-term health problems. Potentially useful questionnaires were discussed by a group of three acupuncturists, all of whom had previous experience of outcome research.

### Evaluating the questionnaires

This study evaluated the two questionnaires identified in the literature review alongside one brief generic questionnaire, the Arizona Integrative Outcome Scale. The analysis explored their ability to reflect the changes in self-concept that were reported in interviews with acupuncture patients and practitioners. The Arizona Integrative Outcome Scale, one-month version (AIOS), that asks for a single VAS score in response to '*Please reflect on your ****sense of well-being***, *taking into account your physical, mental, emotional, social, and spiritual condition ****over the past month'***. It was included as a generic comparator and chosen because it is a new scale that has been developed specifically for use in integrative medicine but sensitivity to change is yet to be established [47,48].

#### a) Recruitment and questionnaire administration

The study was advertised to all patients who were starting treatment with one of five experienced acupuncturists who were registered with the British Acupuncture Council and practice acupuncture in the context of Chinese medicine in private practice in South West England. All the acupuncturists were English. Inclusion criteria were being over 16 years of age; attending for a new course of treatment (i.e. have not attended in the last three months); and having a sufficient level of the English language. The researcher contacted all patients who expressed an interest in the study to answer questions and obtain informed consent, and then posted them the W-BQ12 and AIOS to complete and return. Consecutive responders were recruited until a minimum of twenty participants was enrolled. Three months after starting treatment, just prior to the patient interview the W-BQ12, AOIS and PEI questionnaires were posted out again. No other quantitative data, such as socio-economic status was collected. The study conformed to NHS ethical committee guidelines but was given written exemption from seeking formal approval (because of no connection with the NHS) by the chair of South Western MREC.

#### b) Patient interviews

Three months after starting their acupuncture treatment the participants were interviewed in their own home. The semi-structured interviews were audio taped and transcribed. The interview started by exploring the patients illness and treatment experience, using open questions and prompts that encouraged the patient to talk about their experience in their own words and framework. The second part of the interview used cognitive interview techniques to discuss the patient's responses to the outcome questionnaires. This technique has been used in previous studies [9] and is similar to that recently described as 'Questerviews' [49].

A constant comparative method of analysis was used, in which analysis and interviews are carried out side-by-side, one feeding into the other. Reflexive memos were written throughout the project, and emerging hypotheses were checked out in future interviews. The interview data was coded using a descriptive framework that was developed in a similar previous study and adapted for the present study by adding, removing, or redefining categories until all the interview data could be coded into unambiguous categories. A second, more conceptual, level of coding was developed and applied to all data that had been coded as 'effects of acupuncture'. Within this second level coding was the category 'changes in self-concept'. In addition, within-case analyses summarised each individual account as a vignette (a summary of the account written in the first person and using the participants words as much as possible). All names were replaced by pseudonyms.

#### c) Acupuncturist interviews

At the end of the study each acupuncturist was interviewed to explore the practitioner perspective on what changes the patients reported to them and what they observed themselves. The semi-structured interviews were taped and transcribed and a summary of the data for each participant was made and used in the final analysis.

#### d) Final analysis

The analysis reported in this paper was primarily an evaluative analysis that focuses on the performance of the questionnaires. The final analysis used matrices (table formats) to summarise the data from the different sources and systematically ask questions of the data and look for negative cases. For each patient the response to each questionnaire item and its change over time, were tabulated and compared with the treatment effects as expressed in the patient interviews. The similarities and discrepancies between questionnaire and interview data were considered in the light of the discussion of the questionnaires during the second part of the patient interviews and the data from the acupuncturist accounts. The data was also examined using simple descriptive statistics and correlations. This paper reports on the analysis of the data relating to change in self-concept.

#### e) Minimal important change for the questionnaires

The analysis required some estimation for each questionnaire of what change in score constituted a 'real', or clinically significant, change. Rather than make a qualitative judgement that, say, if the questionnaire scores only changed one or two points that would be labelled as no change, an attempt was made to use the literature to estimate a minimal important change. None of the questionnaires came with guidance on this issue, but papers describing change scores in other populations were available.

The W-BQ12, used in the UK with people with diabetes, demonstrated a within group change of 3.4 and a between group change of 3 and these were statistically significant, and mirrored other outcome measures [50]. Consequently I have taken a change of 3 as the minimal important change.

Studies using the PEI in different patient populations in the UK have found mean enablement scores of 5.39 among acupuncture patients [45], 3.1 among general practitioner patients [51], and 4.7 among homeopathy patients [44]. Consequently I have taken a change of 3 as the minimal important change.

The AIOS is a standard VAS scale. Mean (SD) scores in different populations were 32.53(23.77) for patients in a rehabilitation ward, 60.58 (20.36) for their carers, and 65.8 (19.7) for students. However no studies reporting change scores have been published. I have taken a change of 10 mm to be a minimal important change.

### Calculation of sensitivity and specificity of the questionnaires

Using these minimal important change scores, each participant was categorised as showing no change or a positive or negative change on each questionnaire. For each questionnaire this was cross tabulated with a qualitative assessment of change derived from the interview data and standard methods used to derive specificity and sensitivity. The correlation between the change scores of pairs of questionnaires was calculated (Pearson's coefficient). In view of the small numbers, this numerical assessment is useful as a description but should be viewed as having a wide margin of error.

## Results

These results are reported in two sections. Firstly the results of the literature search and secondly the evaluation of three chosen questionnaires.

### Literature search

These are described under three headings: papers that contributed to the meaning and definition of self-concept; papers describing questionnaires that had some relevance but were not appropriate for the final pilot study; and papers relating to the questionnaires chosen for further study. A number of monographs and papers were examined that are not referenced below because they did not add any new relevant material or were purely the source of a primary reference that was then examined.

#### a) The meaning and definition of terms such as self-concept

This was not the focus of the review but several texts and papers defined and discussed the concepts self-concept and self-esteem [10-13]. A commonly quoted definition of self-concept, is "*the sum total of all that a person feels about himself/herself*", which can be subdivided into four compartments: the body self (physical function and body image); the interpersonal self (psychosocial and sexual interaction); the achievement self (job/role function); and the identification self (spiritual and ethical beliefs) [10]. However because these subdivisions have proved difficult to use in practice, Curbow has suggested that *'The self concept is best viewed as a collection of self representations' *some of which will be core conceptions and some will be more peripheral, and that self-concept is dynamic: active, forceful and capable of change [13]. The affective component of the self-concept is self-esteem, which has been described as '*the disposition to experience oneself as competent to cope with the basic challenges of life and as worthy of happiness.'*[14].*S*elf-esteem has two interrelated components: self-efficacy and self-respect. '*Self-efficacy describes an individual with confidence in the function of her mind, in her ability to think and to understand the facts of reality within the sphere of interest and needs ... When an individual experiences self-efficacy she also generates a sense of control over her life, which contributes, to her well-being. Self-respect means assurance of ones value; ones right to live and to be happy.'*[11]

Whetstone & Hansson, investigating the relationship between self-concept and self-care in the context of health promotion, provide a more psychodynamic description of self-concept [15]. They note that self-concept, consisting of *'one's perceptions, thoughts, feelings and conscious beliefs about the self'*, is *'acquired throughout a child's early developmental stages, socialization patterns, educational exposure and significant life experiences'*.

#### b) Questionnaires of some relevance, but not appropriate, or not available, for further study

The literature search revealed a large number of questionnaires that, whilst they apparently covered the concepts of interest, were found on close inspection to be not appropriate or not available. The reasons that they were not appropriate included a lack of face validity; a focus on different concepts; designed for different populations, such as life-threatening illness; very negative or distressing wording; too narrow a focus on self-efficacy; and extreme length. Sometimes a questionnaire was described in part but not published and further enquires drew a blank, such as the modified Rosenberg Self Esteem Scale [16], and the Personal Concerns and Goals Assessment [17,18]. Questionnaires could be broadly categorised as follows, although there was some overlap between categories: satisfaction with/orientation of life [19-22]; coping with/adjustment to illness [23-27]; quality of life [28-34], Self-efficacy [21,35-39]; self-concept [15,22]; spiritual wellbeing [18,40,41].

Several questionnaires were considered in more detail but were not chosen for the final pilot. The FACIT measurement system has been extensively tested and used and translated into many languages, and combines generic and specific measures [29]. It has a spiritual measure, FACIT-Sp [40] which includes appropriate questions such as 'I feel a sense of harmony within myself', but it was rejected because overall it appears appropriate only for people with cancer and life threatening illness. Similarly, the thoughtful empirical and conceptual work that has led to the McGill Quality of Life Questionnaire [30,31] is also focused on people with life-threatening illness. The broader quality of life measures such as WHOQOL-BREF [34] and the patient-generated questionnaires SEQoL [33] and PGI [32], all had aspects that related to self-concept, but they were considered too broad in scope (including the environment, finances and social capital) and were long and complicated.

#### c) Questionnaires suitable for piloting as outcome measures for acupuncture

Two questionnaires, the Patient Enablement Instrument (PEI) [42] and the twelve item Well-Being Questionnaire (W-BQ12) [43] fitted the inclusion criteria and had the advantage of brevity and self completion. Patient enablement *'describes the effect of a clinical encounter on a patients ability to cope with and understand his or her illnesses' *[44]and the PEI It has six questions: able to cope with life; able to understand your illness, able to cope with your illness; able to keep yourself healthy; confident about your health; able to help yourself. Each question has four response options: much better/better (questions 1–4) or more (questions 5–6)/same or less/not applicable. The PEI was originally designed and validated as an immediate assessment of the general practice consultation, completed as soon as the consultation ends [42]. Since then it has also been used with homeopathic patients [44] and acupuncture patients [45] but it has not been fully validated in such settings. At the time of this study there were no published studies that used PEI to measure change over a period of weeks and months. In order to explore the use of the PEI to detect changes in self-concept that emerge over several weeks or months, the PEI was adapted by replacing the original wording of '*As a result of your visit to the doctor today, do you feel you are:' *with *'As a result of visiting the acupuncturist over the last few weeks or months, do you feel you are:' *The 12- item Well-being Questionnaire (W-BQ12) is an outcome measure that is geared to people with long-term illness and it has three subscales covering energy, positive wellbeing and negative well-being [46]. The positive wellbeing questions relate to self-concept, such as 'I have been happy, satisfied, or pleased with my personal life' and all the questions are scored on a four-point scale anchored with 'all the time' and 'not at all'.

### Evaluating the questionnaires

#### Participants

Recruitment took place over a five-month period in 2003/4. The five acupuncturists handed out 65 information sheets; 27 of these were returned; 25 consented and completed the first set of questionnaires (I ineligible, 1 withdrew); and 23 completed both sets of questionnaires and the interview (one withdrew due to illness, one couldn't be contacted). These 23 participants were all white Europeans, between 22 and 82 years of age and 16 were women and 7 were men. People sought acupuncture for a variety of problems including musculo-skeletal problems such as back, knee, hip, shoulder, elbow pains; hot flushes and period problems; psychological and emotional problems; acid stomach; eczema, asthma, and several less clearly defined conditions. Half of the participants had problems of over five year's duration. Two people had reduction of medication as their main aim, and for many others avoiding or reducing medication or surgery was an important factor.

The interviews lasted between 40–80 minutes and they were held between 10 and 20 (median 14) weeks after the start of their acupuncture. During this time the participants had between one and 15 acupuncture consultations and 13 of them were still having acupuncture at the time of the interview.

#### Changes in self-concept

The qualitative analysis of data has been supplemented at times in this paper with an indication of the numbers of participants in certain categories (added in brackets) and in one case with a statistical correlation. These numerical descriptors are useful as a guide to the spread of the data but as the sample for this study was a convenience sample rather than a representative sample, such numbers cannot be used to generalise in a quantitative sense to any wider population.

Analysis of the interview data provided evidence of some change in self-concept in over half of the participants (15) and this included one or more patients of each of the acupuncturists.

**Figure 2:**
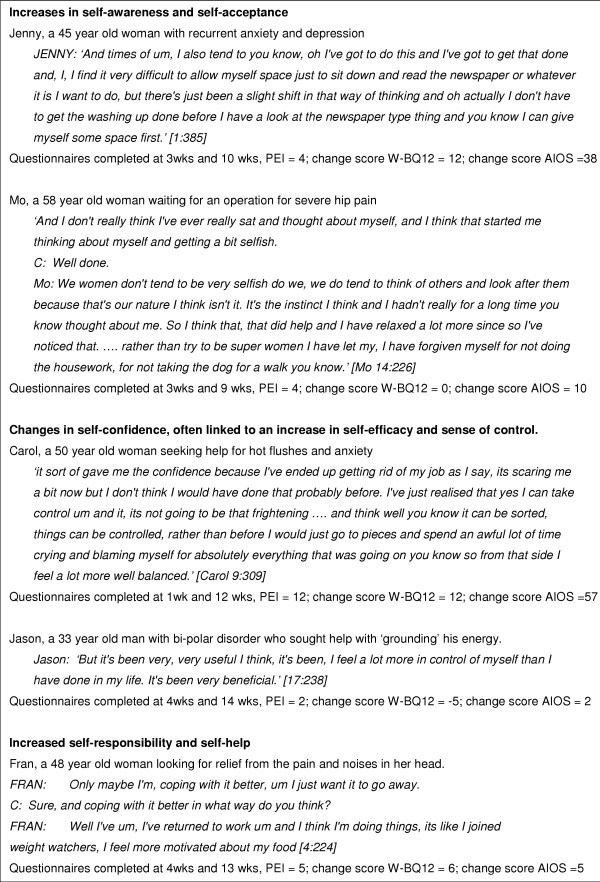
Examples of changes in self-concept. [Questionnaire scores provided as weeks after start of acupuncture and as a positive score = improvement]

Changes were noted across the whole range of self-awareness, self-acceptance, self-confidence, self-responsibility and self-help. Increases in self-awareness and self-acceptance included becoming more aware of the triggers to headaches and stomach problems and being able to accept one's own needs, such as the need for rest. Changes in self-confidence, often linked to an increase in self-efficacy and sense of control, included confidence to seek a new partner, to cope with or to change jobs, or to assert oneself within a relationship. Increased self-responsibility and self-help led to taking more care with diet, taking more exercise and giving themselves more time to relax. Examples of these aspects of self-concept are given in Figure 2.

People were more likely to experience changes in self-concept if they had continued with acupuncture for six or more appointments. Although changes in self-concept were usually associated with improvement in the original symptoms or reason for going to acupuncture, this was not always the case (such as Fran, see Figure 2).

#### Questionnaire performance

The performance of the questionnaires in relation to detecting and measuring these changes in self-concept was evaluated primarily by a qualitative analysis, supplemented by some simple quantitative analysis when appropriate. The first time the questionnaires were completed was between one and four weeks after the start of the acupuncture and the second time was between 10 and 20 weeks after the start of their acupuncture. This variability is taken into account in the qualitative and combined analysis but limits the accuracy of the quantitative data and consequently mean change scores for the questionnaires are not presented. **Each of the questionnaires was incorrectly completed, or refused, by one or more participants**.

The sensitivity and specificity to changes in self-concept was calculated for each of the questionnaires (Table 1) The PEI-ac and the W-BQ12 questionnaires given in detected all but two (out of 15) of the patients who had changes in self-concept at interview. Quantitative tests of correlations between the change scores for each of the questionnaires showed significant correlations between the three questionnaires: PEI and the WBQ-12 (.567); PEI and the AIOS (-.462); AIOS and the WBQ-12 (.537).

**Table 1:** Sensitivity and specificity of the questionnaires: correlation between the interview data and the questionnaire data, n = 23 [change in questionnaire score refers to a positive change of at least the minimal important difference, as described in methods section]

	**Sensitivity **(proportion of patients whose interview data does indicate changes in self-concept that show a change in questionnaire score)	**Specificity **(proportion of patients whose interview data indicates no changes in self-concept that don't show a change in questionnaire score)
Wellbeing questionnaire, W-BQ12	66%	75%
Patient enablement instrument, PEI	80%	88%
Arizona Integrated Outcome Scale, AIOS	60%	33%
One or both of the W-BQ12 and PEI	87%	63%

In order to gain more in-depth understanding of the differences between these three questionnaires, qualitative negative case analysis was used to explore each case in which these three questionnaires did not all show the same change. All of these individual in-depth analyses, along with the data from all the direct discussions about the questionnaires that were part of the interviews, were used to produce a summary of the strengths and weaknesses of each of the questionnaires

## Strengths and weaknesses of the questionnaires, for measuring changes in self-concept

### Patient Enablement Instrument, PEI

#### Weaknesses

• A few people were using acupuncture alongside several other interventions and had difficulty attributing change to acupuncture itself (the question asked is '*As a result of visiting the acupuncturist over the last few weeks or months, do you feel you are:'*).

• A few people felt the questions were not relevant to their aims and experiences of acupuncture and would not engage with the questionnaire. These people either put N/A or 'the same' for all questions, or left it blank. For some of these people there were suggestions elsewhere in the interview that this perspective might change with further treatment.

• Several people commented that the word 'illness' was at odds with the reason they were having acupuncture. For example they viewed their hot flushes as just a sign of aging, or were seeking a more general boost to energy.

#### Strengths

• The questions were simple and straightforward, and relatively unambiguous.

• The scores were reasonably independent of other life events that would affect wellbeing more generally. For example high enablement could occur at the same time as emotional turmoil around a marriage break-up.

• Positive enablement could be scored independently (to some degree) of a deterioration, or lack of change, in physical health. However better physical health usually had a direct enabling effect.

• The explicit timescale 'As a result of visiting the acupuncturist over the last few weeks or months' was interpreted as directing an overall assessment that was not affected by the most recent ups and downs.

### Well-being Questionnaire, W-BQ12

#### Weaknesses

• A number of people wanted more response options, especially as the questions were quite complex and subtle. (However one person liked to have few choices)

• Individual criticism of the wording of many of the questions included:

◦ Crying was not always a negative sign

◦ Afraid 'for no reason at all' didn't apply to them, but wanted to show they were worried (e.g. a woman with cancer)

• A static score often hid changes in subscales, such as positive wellbeing improving but energy worsening due to poor sleep. Reporting the separate dimension scores may therefore be important.

• Ceiling effects in negative and positive wellbeing were found on several occasions. In these instances benefits relating to self concept were not picked up.

• The timescale of 'the past few weeks' made scores susceptible to being swayed by recent events and it didn't sit easily with the positive wellbeing questions.

• Increased self-awareness could lead to an apparent worsening of positive wellbeing

#### Strengths

• Many people found the questions straightforward and preferred them to the VAS style of the Arizona Integrative Outcome Scale. Even those people who found the positive wellbeing questions difficult or seemingly irrelevant usually completed them thoughtfully.

• The three dimensions were clear and their separate changes were meaningful in the context of the interview material

• The scores were able to demonstrate good wellbeing even when physical disability was present

• The four positive wellbeing questions were relevant to many people and showed up changes even in the less reflective respondents.

• There was only one partial non-completion and the lack of N/A option forced choices that appeared relevant on analysis

### Arizona integrative outcomes scale

#### Weaknesses

• Changes in these scores did not enable any distinction between changes in physical, emotional, social or spiritual wellbeing

• Some people found it difficult to average out all of these, especially if changes were occurring in several dimensions, some good and some bad.

• Some people thought their score would not be reproducible, and analysis found one instance of a change in score that was not reflected in the interview data.

• Subtle changes in self-concept were sometimes lost amongst other changes in physical health and life events.

#### Strengths

• The VAS line allowed for an instinctual approach to scoring, which suited some people better than choosing a number.

• It was very quick to do (for most people)

• Some people appreciated having the spiritual and emotional aspects taken into account and thought that giving a combined score made sense and could be done.

• The timescale of one month was useful in people who had varying symptoms, although some people found it difficult to think back that far and focussed more on their present health

## Discussion

### Methodology

The topic of defining, understanding and measuring change in the many aspects of self-concept is a complex one, and a single study such as this can contribute only one part of the whole jigsaw. Despite the thorough and methodical nature of the literature search it is likely that it did not find all the validated questionnaires and it is hoped that others can build on this to make it more complete. The three questionnaires that were further evaluated were chosen on the basis of prior research for a particular patient population, and as such should not be taken to dismiss other questionnaires or preclude their testing and use in future studies. Future plans include a quantitative study using a larger sample of patients.

Notwithstanding these limitations, this study attended to many of the quality issues for qualitative research. The sampling strategy achieved a sample with both men and women and a wide range of age, long-term condition and practitioner. The researcher built on previous experience in the field, and the inclusion of interviews with the acupuncturists brought a wider perspective to the analysis. The data analysis included systematic coding of the data, reflexive and analytic memos, and the use of negative case analysis. The final stages of analysis took the form of checking hypotheses suggested by a quantitative method by returning to the qualitative data, and vice versa. This combination of methods and perspectives helps to overcome the limitations of the small sample and produces findings that are more transferable to other contexts.

### Findings

The three questionnaires had different strengths and weaknesses in relation to measuring changes in self-concept. As expected the generic Arizona Integrative Outcomes Scale (AIOS) lacked specificity and one participant found the VAS scale confusing, but this study confirmed its face validity and is the first to demonstrate its use in measuring change over time. The Patient Enablement Instrument (PEI) was both sensitive and specific in measuring these changes but had lower acceptability. In this study the PEI was slightly adapted in order to ask participants to assess their change over a three month period. In this respect the results contribute to validating its use as an outcome measure beyond the immediate outcome of the consultation. Theoretical concepts about measuring change retrospectively suggest that such assessments are likely to be inaccurate, and the fact that this did not emerge as a major problem in this study may relate to the fact that the 'gold standard' was a single qualitative interview that also assessed change retrospectively. The PEI has recently been successfully used to track changes over time (3 months and 12 months) in patients attending the Glasgow Homoeopathic Hospital, [1. Bikker AP, Mercer SW, Reilly D. A pilot prospective study on the consultation and relational empathy, patient enablement, and health changes over 12 months, in patients going to the Glasgow Homoeopathic Hospital. J Alt. Comp. Med. 2005, 11 (4), 591–600] and these authors report that enablement at contact consultation (the validated use of the measure) predicted ongoing enablement over time. There is therefore some preliminary evidence that PEI may prove useful as an outcome measure beyond the immediate consultation.

The Wellbeing Questionnaire (W-BQ12) is a more traditional outcome questionnaire. The sensitivity of the Wellbeing Questionnaire (W-BQ12) for detecting changes in self-concept was affected by initial high scores (ceiling effect) and a shorter timescale but the questionnaire was acceptable and is suitable for repeated administration. The fact that the W-BQ12 also has a subscale for energy increases it's usefulness in situations, such as acupuncture, where this is an important outcome. The energy change data is not reported in this paper but it broadly supports other work that shows the subscales to be meaningful, relevant and reliable [43]. In situations where both PEI and W-BQ12 can be used, the combination provides many of the advantages and less of the disadvantages of using either one by itself. For example individuals who found the PEI unacceptable were able to complete the W-BQ12, and individuals who had a ceiling effect on the W-BQ12 were able to reflect their changes using the PEI. The fact that changes in self-concept were associated with having six or more sessions of acupuncture and that increased self-awareness could lead to temporary worsening of wellbeing both highlight the importance of measuring such outcomes over a long period of time.

## Conclusion

This study confirms the finding that changes in self-concept are one of a range of outcomes experienced by people having acupuncture and Chinese medicine in the UK. The literature search revealed a large number of measurement scales for certain aspects of self-concept in different patient populations, such as people with cancer, diabetes and other long-term illness, and two of these questionnaires were assessed as potentially applicable for this patient population. The subsequent in-depth mixed methods evaluation of these two questionnaires, alongside a new generic outcome scale, largely confirmed their usefulness in the context of acupuncture and Chinese medicine as well as providing more transferable information about their strengths and weaknesses. These findings are now available for researchers to use when choosing outcome questionnaires for other interventions.

Most striking of all in this study was the emergent and complex nature of these changes in self-concept, the difficulty that some interviewees found in articulating such changes, and the fact that for some people these were the only benefits they experienced. This confirms the importance of including these outcomes in evaluations of complex interventions and of following up participants for six or twelve months. The results of the in-depth evaluation of the three outcome measures demonstrate how thoughtful and focused we need to be in choosing the best tool for the particular intervention and population. Qualitative methods provide in-depth information about how people interpret and score questionnaires, and results that can be useful to researchers in other fields. The Wellbeing Questionnaire W-BQ12 and the Patient Enablement Instrument both show promise for measuring changes in self-concept and further work is planned. It is hoped that this paper provides a basis on which to build more collaboration and work in this area, across a wide range of long-term health problems and questionnaires.

## Competing interests

The author(s) declare that they have no competing interests.

## Authors' contributions

CP conceived, designed and carried out the study, and wrote the manuscript.

## Pre-publication history

The pre-publication history for this paper can be accessed here:


